# Synthesis of Scandium Phosphate after Peroxide Assisted Leaching of Iron Depleted Bauxite Residue (Red Mud) Slags

**DOI:** 10.1038/s41598-019-48390-z

**Published:** 2019-08-14

**Authors:** Bengi Yagmurlu, Gözde Alkan, Buhle Xakalashe, Claudia Schier, Lars Gronen, Ichiro Koiwa, Carsten Dittrich, Bernd Friedrich

**Affiliations:** 1 IME- Process Metallurgy and Metal Recycling, RWTH Aachen University, Aachen, Germany; 2 MEAB Chemie Technik GmbH, Aachen, Germany; 3 IML- Chair of Applied Mineralogy and Economic Geology, RWTH Aachen University, Aachen, Germany; 4 College of Science and Engineering, Kanto Gakuin University, Yokohama, Japan

**Keywords:** Chemical engineering, Chemical engineering, Sustainability

## Abstract

Anticipated future demand and limited primary sources of Sc highlight the importance of secondary Sc resources such as bauxite residue (red mud). In this study, a process route starting from red mud aiming to recover Sc as a concentrate by a combination of pyrometallurgical and hydrometallurgical processes was developed. Bauxite residue was treated in an electric arc furnace (EAF) for Fe removal as well as slag conditioning with varying flux additions and various cooling conditions. 95% of iron recovery to the metal was achieved. Resulting slags were subjected to identical H_2_O_2_ supported H_2_SO_4_ leaching conditions at 75 °C. The effect of slag mineralogy and crystallinity on the leaching efficiencies were investigated using XRD and QEMSCAN analysis. As a result of the highly amorphous nature of acidic slags, maximum of 72% Sc leaching was obtained. For leached slags, water quenched basic slag was found to be the most promising condition resulting in an extreme Sc leaching yield of 97% and this slag was selected for the further Sc precipitation. High impurity removal rates and selective Sc separation were achieved with a triple-stage successive precipitation to synthesize a Sc concentrate. Starting from EAF treatment followed by leaching and precipitation, 85% of the initial Sc in the red mud was successfully recovered as Sc phosphate.

## Introduction

Scandium (Sc), which is used as a tuning metal in Al-Sc alloys to enhance their strength^1,2^, weldability^3^, heat and corrosion resistance^4^ as well as in solid oxide fuel cells (SOFCs) to achieve extreme oxygen-ion conductivity, is the focus of great interest^5,6^. However, due to the geographical scarcity of primary resources and complex extraction and purification routes of secondary resources^7^ such as uranium^8^, titanium pigment^9^ and nickel laterite processing^10,11^, Sc in metallic form is extremely expensive^12^. The current global Sc production is approximately 15 tonnes^13^; however, the demand is foreseen to upsurge as a result of the technological developments, especially in the energy field (i.e. SOFCs). In order to prevent a possible future shortage caused by produced vs demanded amount or political conflicts and increase Sc supplies at reasonable prices for future demand, new resources with feasible extraction techniques must be investigated.

Bauxite residue, which is the residue of Bayer process, contains between 15 mg/kg and 170 mg/kg Sc depending on the bauxite source^14–17^. When the excessive global production rates of red mud (~150 million ton) are considered, that amount of Sc is highly promising as a possible resource. Different techniques based on hydrometallurgical processes for the Sc recovery had been studied before with low solid to liquid ratios (e.g. 1/50)^18,19^ and pre-treatments such as microwave^20^ to achieve higher leaching efficiencies. In these studies, by the help of extreme leaching conditions (high acid and energy consumption), substantial leaching efficiencies up to 70% of Sc were reported, however, with low selectivity over iron which is one of the main elements in bauxite residue. In our previous study, we utilized a novel H_2_O_2_: H_2_SO_4_ acid combination to promote the oxidative nature, which ensured low solubility of Fe and precipitation of Si to handle the Si-gelation problem^21^. Although hematite dissolution is supressed to a certain extent, Sc is mainly hosted by hematite^22^ and thus, there was still “Fe” which acts as the major impurity in pregnant leaching solution (PLS), pointing to the use of Fe depleted slags as a promising alternative.

Considering these issues, a complete process was designed in lab scale with the aim of scaling up to pilot scale as future work. The proposed process is starting from red mud, recovering iron as pig iron and concentrating Sc in the resulting slag via smelting, followed by hydrometallurgical treatment of the separated slag ending up with a Sc enriched PLS and finalized by selective precipitation of the Sc concentrate.

Fe recovery from red mud can be achieved by electric arc furnace (EAF) smelting yielding Fe recoveries of 95% with pig iron as the main product and a slag enriched in Sc, Ti and REEs with desired crystallinity and chemistry as the by-product. The type and amount of fluxing agents added during smelting and the cooling rates after smelting have a crucial role in the conditioning of the produced slags^23–25^.

In this study, a total of six different Fe depleted slags were investigated with three different chemistries from acidic to basic and two different crystallinities for each slag chemistry classified by cooling rates. For all investigated slags, oxidative H_2_O_2_:H_2_SO_4_ combination was used as leaching solution. The effect of chemistry and crystallinity on leaching efficiencies as well as kinetics for Sc were investigated by inductively coupled plasma (ICP), X-ray diffraction (XRD) and QEMSCAN methods. Moreover, the strength of this combination on the prevention of silica gelation was assessed with high Si containing slags. The most promising slag was used for further purification and precipitation steps; in these cases almost complete Sc precipitation was achieved. The proposed route and the summary of each step was given in Supp. Fig. S1.

## Results and Discussion

### Slag design

**Figure 1:**
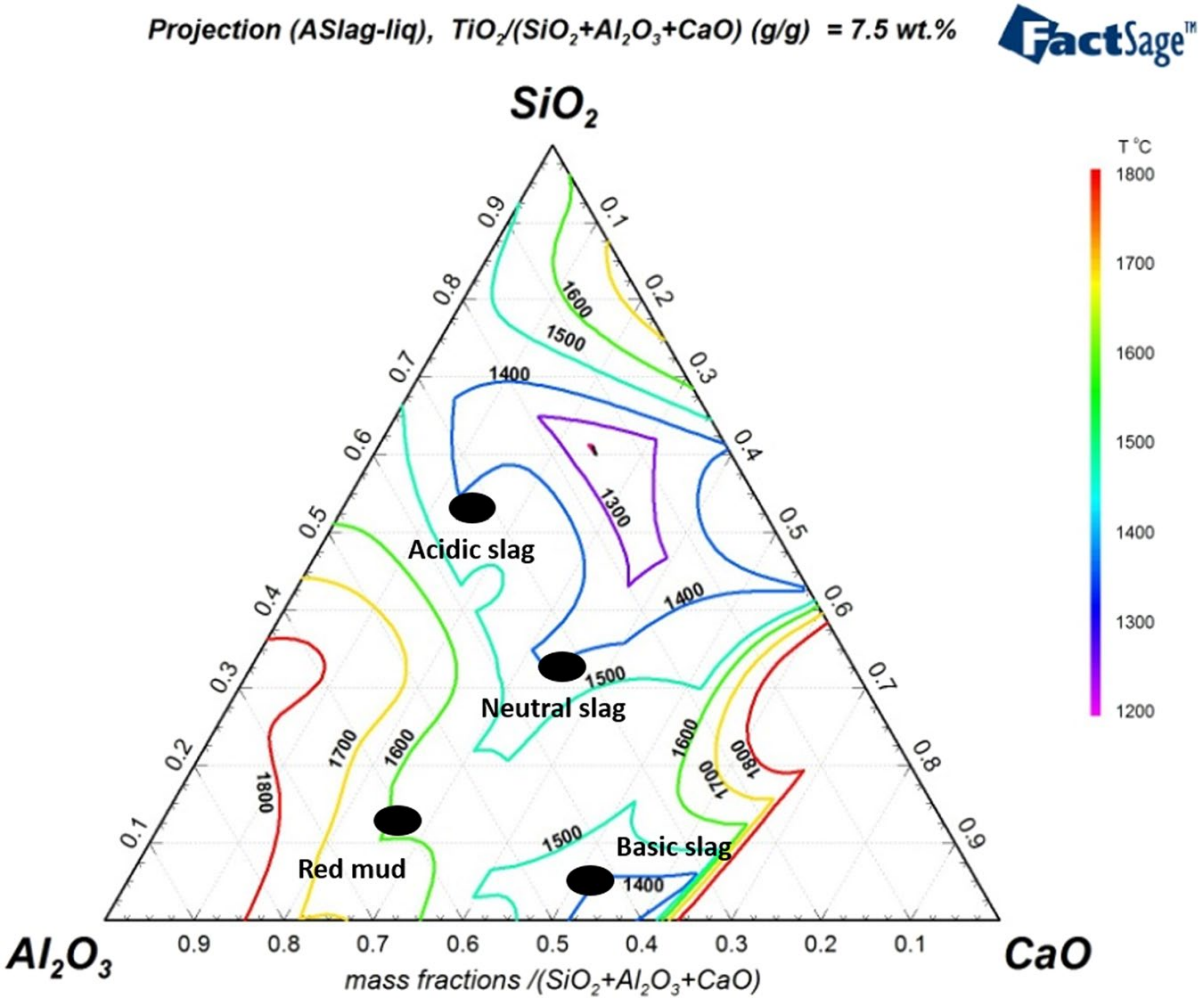
Ternary phase diagram for the Greek red mud and synthesized slag system.

Direct carbothermic reduction of red mud requires high smelting temperatures above 1600 °C (Fig. 1). This in turn demands high-energy input into the smelting process; furthermore, the corrosion of refractory material during smelting can be increased under these conditions. Moreover, high alumina slags which are produced through direct smelting of red mud are highly viscous and this could lead to metal entrainment in the slag, therefore additives during the smelting process are necessary^26,27^. A fluxing strategy was employed to achieve low melting slags and to avoid the presence of a solid slag fraction during smelting.

For favourable leaching of the valuable elements, efficient removal of Fe from the slag during the smelting process is necessary^28^.

**Figure 2:**
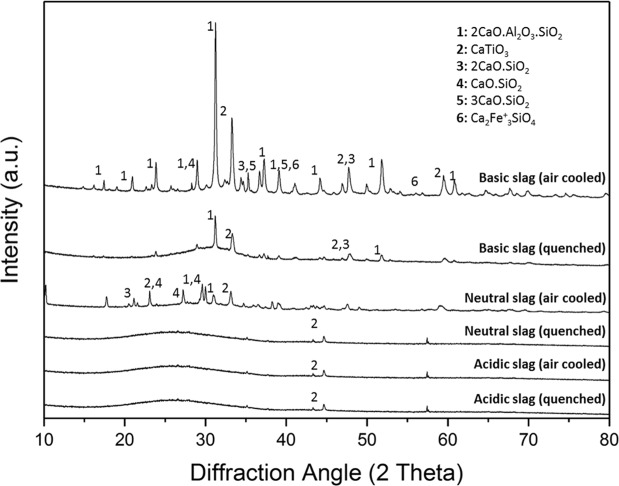
XRD analyses of all examined slags.

On the basis of the design criteria stated above, three different chemistries of slags were designed; a basic slag by lime fluxing, a neutral slag by a combination of lime and silica fluxing, and an acidic slag by silica fluxing as shown in Fig. 1. Furthermore, each of these slags was cooled under ambient conditions and under water quenching to achieve different degrees of crystallinity. The XRD diffractograms of these six slags are given in Fig. 2.

The basic slags prepared with 20 wt.% lime fluxing were liquid at the operating temperature of 1500 °C. Higher amounts of lime addition were not considered since this would require more energy for processing and could also lead to an increased melting point of the slags. The most predominant phase in the basic slag cooled under ambient conditions was found to be gehlenite (2CaO.Al_2_O_3_.SiO_2_) as can be seen from Fig. 2. It is important to note that the Ti containing phase was detected as perovskite in this slag. The quenched slag exhibited a glassy structure where the phase formation was incomplete or minimal.

The neutral slags (10 wt.% lime and 10 wt.% silica to red mud) were molten below the operating temperature of 1500 °C. Higher additions of lime and silica were predicted to produce an even lower melting slag, but such additions were not considered so as to avoid dilution of valuable elements in the slags^29^. The most predominant phase in the neutral slag cooled under normal conditions was revealed also as gehlenite. It is important to note that the Ti containing phase was again found to be perovskite in this case. The quenched slag sample also shows a glassy structure.

The acidic slags were generated by additions of 20 wt.% of silica to red mud. The liquidus temperature of these slags was below 1500 °C. Higher additions of silica were predicted to produce a highly viscous slag, which would make the slag metal separation a challenging process. The acidic slags in both cooling conditions were found to be highly glassy, especially in the case of the quenched slag (Fig. 2). The perovskite phase was minimal in these slags due to the lack of crystallization.

Moreover, it is worth to emphasize that for all slags, Fe recoveries were more than 95%. This is also a validation that the 10 wt.% of lignite coke addition during the smelting of red mud was sufficient for the reduction of iron in red mud. Furthermore, co-reduction of V, into the pig iron product was achieved, with reported recoveries greater than 80%. This is attractive for the smelting process as V is classified as a critical element like Sc, and V can be recovered from the produced pig iron^13^.

**Figure 3:**
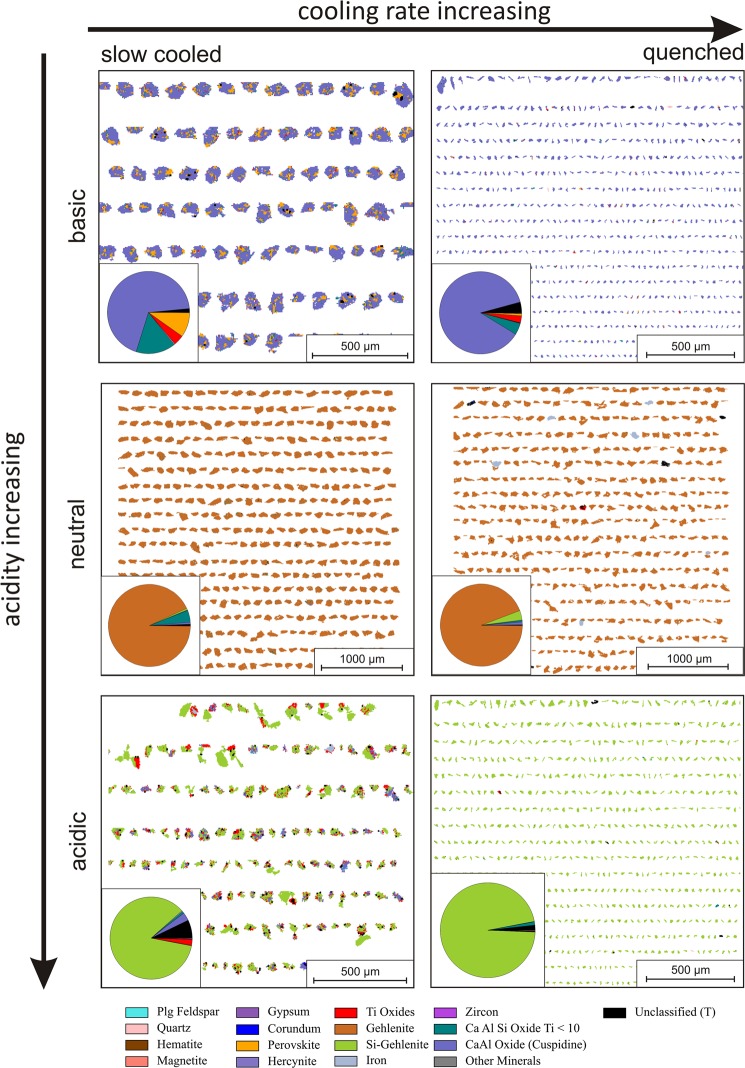
Mineralogical distribution of the six synthesized slags with various acidities and cooling rates by QEMSCAN analysis, in which cooling rate is increasing horizontally and acidity of the slag is increasing vertically.

Since most of the slags exhibited highly amorphous nature and difficult to characterize solely by XRD, QEMSCAN analyses was performed to obtain detailed insights from mineralogical distributions and association, which directly affects the leaching behaviour. Figure 3 shows the mineralogical distribution of the produced slags. The quantitative ratio of the mineralogical phases in each slag was given in Supp. Fig. S2.

Mineral mapping and distribution of the slags indicates a gradual change from acidic to basic slag. QEMSCAN reveals that the minerals associations in all slags are complex, they mostly entrap each other, and therefore hard to be detected by XRD. The main mineral of basic slag is reported as cuispidine which is a CaO enriched Ca-Al-Si oxide. Cuispidine replaced with gehlenite, which is also a Ca-Al-Si oxide with different stoichiometry, in the case of neutral slag and with Si enriched- gehlenite in the case of acidic slag with more addition of SiO_2_, which are consistent with the XRD findings. Moreover, Ti was detected as perovskite in air cooled basic slag, which also gradually changed into TiO_2_ with the decrease of CaO content of the slag.

Independent from the chemistry of the slags, air cooled and quenched slags exhibited apparent difference in particle size. Coarser particles of air cooled slag were replaced with finer ones by quenching, which may be due to the rapid solidification of the slag and lack of crystal formation and growth. Moreover, in quenched samples, secondary minerals associating with the main one were hardly detectable again emphasizing lack of crystallization and highly amorphous content of quenched slags, in agreement with XRD findings.

### Effect of slag chemistry and cooling rates on peroxide assisted leaching

Due to the enrichment of SiO_2_ content in all slags, the risk of gelation may be even more pronounced. That’s why the previously developed novel H_2_O_2_ assisted leaching process was applied, which induces the precipitation of the dissolved Si as SiO_2_^21^. Furthermore, Sc is mainly hosted by hematite in the case of red mud, but it was found that Sc is mainly located in Al related phases and some minor amount is in Ti related phases^22^. Hence, leaching should target those minerals to achieve high Sc leaching efficiencies.

Fe was not included in the calculations since some minor metallic Fe, which could be leached out very easily, was detected in the analysis. Hence, the calculations based on mass balance disregarding the metallic Fe could be misleading.

#### Leaching of acidic slags

**Figure 4:**
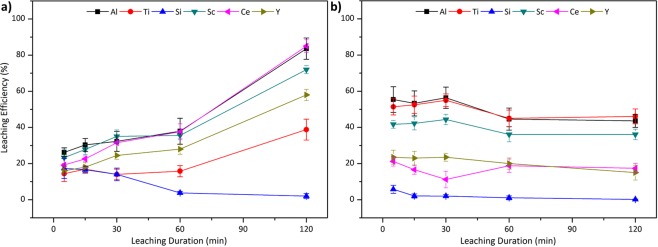
Leaching efficiency of Al, Ti, Si, Sc, Ce and Y from (**a**) slow cooled and (**b**) fast cooled acidic slag with 2.5 M H_2_SO_4_:2.5 M H_2_O_2_ mixture at 75 °C with S/L = 1/10.

Figure 4 shows the leachingefficiencies of the constituent elements from air cooled and quenched acidic slags.

It can be seen from Fig. 4 that the leaching behaviour is differentiating from each other although the slags were only subjected to different cooling conditions. The major reason for this difference is the various crystallinity of the slags as well as the fluctuations on the mineral structure of the slags. It was stated earlier that, addition of a glass-forming compound, SiO_2_, resulted in highly amorphous structure in the slag, which directly affects the leaching efficiencies. In addition, Al and Ti phases, which are the main host phases of Sc, are associated with the Si phases in the acidic slags. That is why the structure of the phases found to be directly related with the efficiency of Sc.

As it can be seen in Fig. 4(a), when acidic slag is poured into a refractory lined mould for slow cooling, efficiencies were observed to be stable until 60 min leaching duration. After that point, almost all of the elements showed an increase in the leaching efficiency. Even though this slag was slow cooled, it is still highly amorphous. Additionally, QEMSCAN investigation on the mineral distribution showed that, Si-gehlenite entrapped the major minerals inside the particles while having larger particle sizes, and relatively smaller free surfaces, than the identical slag subjected to higher cooling rates. Therefore, at first, the acid reacts with the available free surfaces, while it took longer time to penetrate into this Si-gehlenite shielding, which delays the leaching process. Despite high Al leaching efficiency, around 80%, due to dissolution of Si-gehlenite shielding and CaAl-oxide phases over time, low Ti leaching was achieved due to the placement of Ti in the amorphous phases as well as insufficient penetration of the leach solution into the particles limited the leaching efficiency of Sc. Maximum Sc leaching efficiency of 72% was reached after 2 h of treatment.

As predicted, the amorphous nature of the quenched slag as shown in Fig. 4(b) inhibited the leaching reaction beyond at the available surface on the periphery of the slag particles, as these phases tend to have low reactivity with an acid. Contrary to the slow cooled counterpart, this slag offered more free surfaces and less entrapped phases inside the Si-gehlenite matrix. Thus, the acid reacts immediately at the early stages of the leaching with the available phases and could not reach beyond the periphery of the slag particles as a consequence of high amorphosity. Almost no change was observed during 2 hours and therefore Sc leaching rates stayed stable during the whole duration, which is around 50%.

As it was reported in our previous study^21^ dissolved Si is oxidized and re-precipitated in the form of stable quartz in both cases, yielding continuously decreasing Si leaching efficiencies as duration increases.

It is important to note that leaching of slags needs more time than direct leaching of red mud as a result of more stable phases formed by high temperature treatment. Dissolution of the phases continue throughout the leaching duration since the dissolution of the stable crystalline phases and oxides need longer time. Similarly, extensive amorphous content, which makes the extraction more challenging since the penetration of the slag particles by the acid is inhibited, also decreases leaching efficiencies. These point out an optimum content of crystallinity in the slags is needed for the enhanced leaching.

#### Leaching of neutral slags

**Figure 5:**
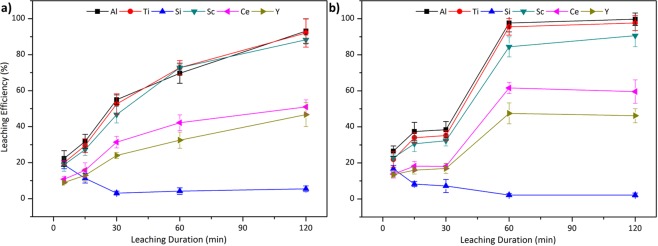
Leaching efficiency of Al, Ti, Si, Sc, Ce and Y from (**a**) slow cooled and (**b**) fast cooled neutral slag with 2.5 M H_2_SO_4_:2.5 M H_2_O_2_ mixture at 75 °C with S/L = 1/10.

Leaching efficiencies of the air cooled and quenched neutral slags are presented in Fig. 5.

In this case, less SiO_2_ was introduced during smelting together with CaO to produce neutral slags, which reduces the amorphous phases as can be seen from the XRD. In the case of slow cooled slag [Fig. 5(a)], leaching efficiencies of the constituent elements continued to rise as the leaching duration increased as a result of relatively higher crystalline structure. As in the acidic slag, the easily accessible phases in the free surfaces reacted and immediately yielding 20% leaching efficiency for major elements. Gehlenite, the major phase found in neutral slags as the slag matrix, surrounded the particles; hence, the leach solution was first reacting with the gehlenite, then extracting the elements from the phases trapped inside the matrix of the slag particles. In addition, since Ti can be found mainly in Ca-Al-Si-Ti-oxide in this slag, Ti extraction was also observed to rise steadily with leaching time.

Figure 5(b) shows a rapid dissolution of the constituent elements upon commencement of leaching, since the quenched slag offers more free surfaces than the slow cooled counterpart does. It can be seen that, after initial reaction, leaching efficiencies were found to be stable for a short time interval until cracks were formed at the surface of the particles increasing the accessible reaction surface beyond the periphery of the slag particles, where a relatively thinner layer of gehlenite shield is formed. Nevertheless, after 30 min operation duration, a sharp upsurge in leaching efficiencies of the targeted elements was detected, since the reaction inside the smaller particles initiated at once. After this point, extraction of the elements reached a maximum at 60 min of leaching and remained stable for the rest of the process. Contrary to the quenched acidic slag case, high leaching efficiencies were observed as a result of the more optimum crystallinity of the phases.

In both cases, high Al, Ti and Sc leaching efficiencies were obtained while Si gel formation is supressed. Since Sc is present in Al and Ti phases, as their dissolution is favoured, Sc leaching efficiency was enhanced as well. Sc leaching efficiencies were found to be 88% for slow cooled slag and 90% for quenched slag. Moreover, REEs extraction was also observed to be lower than acidic slags. One possible reason for lower leaching efficiencies of REEs is the enrichment of the REEs in non-targeted and difficult to leach mineral phases.

#### Leaching of basic slags

**Figure 6:**
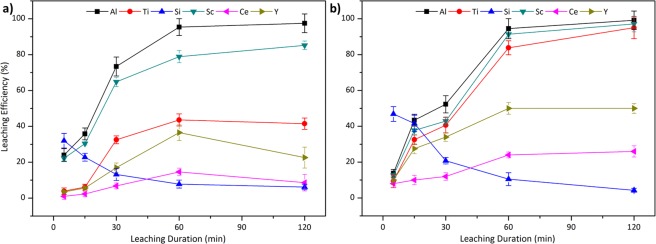
Leaching efficiency of Al, Ti, Si, Sc, Ce and Y from (**a**) slow cooled and (**b**) fast cooled basic slag with 2.5 M H_2_SO_4_:2.5 M H_2_O_2_ mixture at 75 °C with S/L = 1/10.

The replacement of SiO_2_ with CaO resulted in formation of a basic slag and the leaching efficiencies of both slow cooled and quenched basic slags are shown in Fig. 6.

Although introducing high amounts of CaO into the slag has a drawback, as it favours the formation of gypsum when it reacts with sulfuric acid leading to high acid consumption, the leaching efficiencies showed highly promising results. Additionally, this drawback could be controlled by controlling the phases formed in the slag.

The leaching behaviour of the slow cooled slag is presented in Fig. 6(a). Upon addition of the leachate, Ca-related phases directly reacted and produced gypsum and consequently, initial leaching efficiency of Ti was found to be below 5%. Leaching efficiency of Ti increased with time and peaked at 60 minutes of operation duration where Al and Sc efficiencies were found to be 95% and 79%; respectively. After this point, no major change in leaching was observed as Al and Sc reached 98% and 83% dissolution rate, leaching efficiency of Ti remained stable around 40%. Since Ti phases are a minor source of Sc, it is expected to observe lower Sc efficiency than neutral slag. Major Al source, calcium aluminate phases in this case, were dissolved with time that generates high Al extraction yields. On the other hand, QEMSCAN investigations showed that, Ti is mainly entrapped in these calcium aluminate phases. Reaction between calcium aluminates and sulfuric acid yields a gypsum layer and, this gypsum layer acts as a barrier preventing leach solution to reach entrapped minerals which drops the Ti leaching rates. Another reason behind low Ti efficiency could be explained by the formation of highly stable perovskite phase which was not completely dissolved in these leaching conditions. In addition, REEs is found to be related with the Ti dissolution; therefore, it could be suspected that these REEs could be also associated with the formed perovskite.

When the basic slag was quenched, not only the free surfaces for reaction were increased but also the formation of perovskite was suppressed to a certain extent resulting in a positive impact on Ti dissolution behaviour. In addition, owing to finer particles, gypsum formation which behaves as a barrier for further reaction was prevented, and consequently higher leaching yields for target elements were reached. As a result of higher amorphosity, more controlled dissolution than for the slow cooled counterpart was achieved. Similar to the previous quenched slag cases, leaching initiated at the free surfaces with lower rate until partially amorphous calcium aluminates shield surrounding the particle was breached. Thus, the leaching reaction was almost completed at 60 min of leaching and a minor increase in leaching efficiency was observed after this point. The best leaching efficiencies were observed, reaching 99%, 95% and 97% for Al, Ti and Sc respectively, at the end of 2 h.

In both cases, low REEs extraction was observed, since these elements were not target for this study and there was no emphasis on optimising the slag properties and the leaching conditions for their recovery. High Si dissolution at the beginning due to the dissolution of CaAlSiTi-oxide phases were observed for both cases, and dissolved Si in the aqueous solution was precipitated as SiO_2_ thanks to the oxidizing leaching conditions.

### Removal of impurities and precipitation of scandium

After transferring Sc from a solid slag phase to an aqueous solution, recovery operations take place. However, having many impurities in the PLS inhibits easy and low cost purification operations for Sc. In our previous studies, we developed a simple successive precipitation method to synthesize a Sc concentrate to ease the purification operations by concentrating Sc, as well as decreasing the level of impurities^30–32^. This process could be controlled by pH adjustments and proved to be successful to isolate the major impurities while forming a Sc phosphate concentrate (See Supp. Fig. S3). Furthermore, this concentrate is easily soluble in an acidic medium and could be further treated by standard methods much easier than advanced purification operations.

Firstly, the pH of the aqueous leach solution was increased from 0.3 to 3.4 by the addition of NH_3_(aq) aiming to remove the majority of the impurities with minimum loss of Sc. In the 2^nd^ impurity removal step, pH was further increased to 3.8 to achieve maximum impurity removal before main precipitation step. Although the Sc loss is relatively higher in this step, these losses can be disregarded since the residue from this step was recycled into the initial feed. Finally, the pH of the remaining aqueous solution was adjusted to 2.2–2.3 to avoid unwanted hydroxide precipitation upon addition of the dibasic phosphate solution. Immediate phosphate precipitation was observed by the addition of (NH_4_)_2_HPO_4_. The composition of the PLS obtained by leaching quenched basic slag was given in Supp. Table S1.

**Figure 7:**
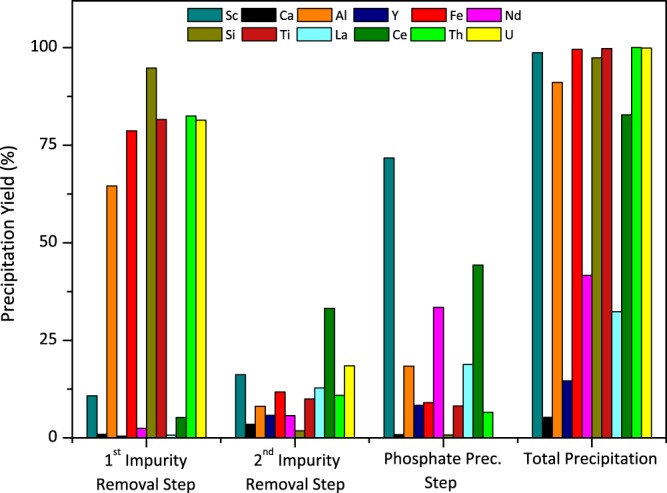
Precipitation yield in each precipitation step of major and minor elements in the PLS.

In the 1^st^ impurity removal step, as can be seen from Fig. 7, significant portions of the major impurities, Fe, Al and Ti are removed with a precipitation yield of 79%, 65% and 82%; respectively. Importantly, only 9% of Sc is co-precipitated, which is the equivalent of 1.5 mg/L in the liquid concentration. Using other hydroxide donors instead of NH_3_(aq) results in high co-precipitation of Sc especially with Fe. However, when NH_3_(aq) is used, special Sc hexamine complex is formed, therefore Sc remained in the solution while impurities were removed^33–35^. Additionally, REEs remained in the solution since their precipitation is not favoured in this pH range. It is important to note that, a majority of the radioactive elements, U and Th, present in the PLS was removed during this step. It is a known fact that these radioactive elements normally follow Sc in commonly preferred purification operations and indirectly become concentrated with Sc^36,37^.

In the 2^nd^ impurity removal step, 12% of the total Fe, 8% of the total Al and 10% of the total Ti as well as 33% of total Ce and 11% of Th were precipitated. In this step, 16% of the total amount of Sc is co-precipitated as well, therefore the precipitate obtained in this step were recycled into the initial PLS to minimize the Sc and REEs losses. Since the amount of Fe and Al are much lower than in the first step, the recycling process does not cause a major upgrade in the concentration of these impurities in the initial feed.

In the phosphate precipitation step, precipitation proceeds in the order of Sc(III)  =  Fe(III) > Al(III) > Ti(IV) > REEs(III)^30^. The removal of Fe from the system beforehand, makes this process very selective for Sc. The elevated amounts Al in the PLS is the main reason why Al is also precipitated and became the major impurity in this concentrate. Nevertheless, separation of Al and Sc is much easier in further purification operations. In this step, 72% of total Sc, 18% of total Al as well as 44% of total Ce and 33% of total Nd were precipitated. Major impurities such as Ti, Fe and Si were found to be less than 8%.

**Figure 8:**
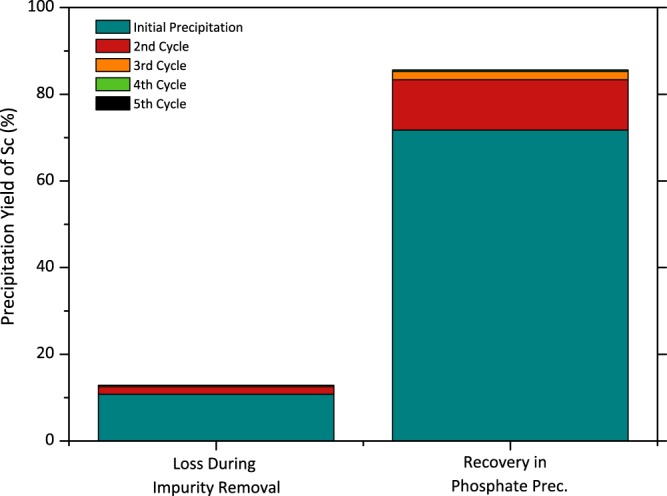
Recycling Sc-containing residue obtained after the 2^nd^ impurity removal step and the variation of the recovery yield.

Although Sc precipitation efficiency was found to be 72%, the major part of the losses, 16% of total content in 2^nd^ impurity removal step, is recycled into the initial feed. Hence, Sc content in the initial PLS and directly the efficiency of the precipitation was enhanced. Figure 8 shows the effect of precipitate recycling on Sc loss and recovery of Sc during phosphate precipitation. As it can be seen from this figure, even though the loss of Sc is increased to 12% and stabilized after the 5^th^ cycle, the Sc precipitation efficiency during phosphate precipitation was increased up to 85%.

In total 99% of Sc, Fe, Ti and Si were precipitated throughout the precipitation process while 90% of Al was precipitated. REEs tends to co-precipitate up to limited extent, but they remained in the solution after this successive precipitation and can be further treated for recovery operations if intended. Since almost all of the major impurities are eliminated, it would be also possible to prefer simple recovery methods for REEs refining. Furthermore, elimination of U and Th during first Fe removal step enhanced both the quality of the Sc concentrate and eliminated the possibility of concentrating the radioactive elements while enabling easy Sc and REEs purification downstream.

## Conclusions

A complete process route starting from red mud until Sc phosphate concentrate was investigated by means of pyrometallurgical and hydrometallurgical methods. Six different slags were produced in an EAF for Fe depletion under different flux additions using lime and silica as fluxing agents and under different cooling rates (air cooled, quenched). Iron recovery to the pig iron product was in excess of 95%, this was achieved with co-reduction of V to the metal in excess of 80% recovery. These slags were subsequently exposed to H_2_O_2_ supported acidic leaching. Depending on the crystallinity and leading minerals in the slag, various leaching behaviours were observed. Increasing SiO_2_ additions in EAF and higher cooling rates of slags result in highly amorphous slags which exhibited limited leachability. Lime fluxed and quenched slag comprised of less crystallized and fine Ca-Al oxide particulates was determined as the most feasible slag for further treatment with promising Sc (97%), Ti (95%) and REEs (50%) leaching efficiencies without silica gelation problem. Pregnant leaching solution of this slag was treated for the further precipitation and purification steps and in total with a 15% loss, 85% of the Sc of red mud was successfully precipitated as Sc phosphate.

The final synthesized concentrate mainly consists of Al, which is easy to separate from Sc, as the major impurity, minor Fe and Ti and most of the Sc from the PLS. Sc is upgraded from mg/L level in PLS to 2–3% in solid precipitate by a simple and pH controlled precipitation route. Therefore, simple purification routes, such as solvent extraction with less stages, could be implemented for further purification of this Sc concentrate owing to the successful separation of major impurities as well as radioactive elements, easy dissolution of the precipitate in diluted acidic conditions and high Sc content.

## Methods

Red mud used in this study was provided by MYTILINEOS S.A. Aluminum of Greece. The dried red mud was subsequently mixed with lignite coke containing 87 wt.% fixed carbon and flux, where the fluxing agents were lime and silica with 95 wt.% CaO and 98 wt.% SiO_2_, respectively. The additions of lignite coke and total flux to bauxite residue were 1:10 and 1:5, respectively. Flux additions of 100% lime, 50% lime and 50% silica, and 100% silica were undertaken. Batch masses of 1.5 kg of the aforementioned recipes were fed into a 100 KVA DC electric arc furnace. The material was contained in a graphite crucible, and the smelting was undertaken at temperatures around 1500–1550 °C, for a holding time of one hour. At the end of each experiment the molten material was poured into one of the two containment set-ups for cooling as follows: a refractory-lined mould where the material cooled down under ambient conditions and the metal settled at the bottom of the mould, or a bucket filled with water for fast cooling of the molten material. Separated slag was then prepared for leaching where it was crushed and milled to obtain a slag fraction of <90 μm.

The treatment of the milled samples for chemical analysis included digestion with a mixture of acids (5 ml HCl, 1.5 ml HNO_3_, 1 ml HF and 3 ml H_3_PO_4_) followed by microwave heating under pressure to temperatures in the region of 240 °C. After the first heating step 5 ml of H_3_BO_3_ was added to the mixture and the sample was heated further in a microwave for 30 minutes to bind free F^−^ ions. The concentrations of the elements of interest were determined by Spectro ICP-OES Spectro Ciros Vision analysis. The chemical composition of red mud and slags evaluated by ICP-OES are provided in the Table 1.

**Table 1:** Chemical composition (wt.%) of bauxite residue and EAF treated slags.

Sample	Fe_2_O_3_	Al_2_O_3_	CaO	SiO_2_	TiO_2_	Sc (mg/kg)
Bauxite residue	43.5	24	10.2	5.5	5.6	120
Basic slag	1.8	38.3	43.2	7.6	7.6	170
Neutral slag	1.5	39.8	29.9	22.0	7.4	170
Acidic slag	1.2	36.8	15.3	38	7.3	170

Particle size distribution was obtained using QEMSCAN based image analysis^25^. Thereby, multi-phase particles where extracted virtually from the recoded field images. Before analysis particles < 10 µm were filtered out to avoid misinterpretations. The generated particle population was analysed for their size distribution by the use of equivalent sphere diameter (ESD) method according to Heilbronner and Barrett^38^.

A typical leaching test was carried out with a glass beaker, heating plate and magnetic stirrer for controlling the reaction temperature and stirring speed. Slags were fed into the reactor, containing preheated H_2_SO_4_ and H_2_O_2_ combination. This mixture were carefully prepared by slowly adding hydrogen peroxide into sulfuric acid to control the rapid exothermic reaction. 2.5 M H_2_SO_4_ - 2.5 M H_2_O_2_ acid combination was used for leaching of all slags at a set temperature of 75 °C, 250 rpm stirring speed, and S/L ratio of 1/10, which were reported as the most promising leaching conditions in our previous study. Leaching experiments were repeated three times independently to ensure the accuracy of the calculations and the analysis. Errors for each sample were calculated, which stayed in the range from 0.8% to 7.9% for all samples. After leaching, samples were vacuum filtered with a vacuum pump and fine filter paper (2–4 µm pore size) to separate solid residue and leachate. Moreover, from ongoing reaction, at determined times of 5 to 120 minutes, samples were collected and immediately vacuum filtered to study the leaching kinetics.

NH_3_ solution was prepared from technical grade (25 wt.%) NH_3_ and diluted until 12.5 wt.% NH_3_ solution was obtained. The precipitation agents were carefully added using a precision burette into 50 mL of PLS while monitoring pH and temperature. All precipitation experiments presented in this study were done at room temperature. For impurity removal step using NH_3_(aq); precipitation solution was added until the target pH was attained under mild agitation. (NH_4_)_2_HPO_4_ solution was prepared as 1 mol/L and added successively after dual impurity removal step. The resulting suspension for each case was then stabilized and homogenized at specified pH for 2 hours and subsequently filtered through fine filter paper. The separated solid residue was washed with distilled water and dried at 110 °C for 24 h.

XRD analyses were performed by the use of a Bruker D8 Advance diffractometer, which uses Bragg- Brentano geometry and θ- θ synchronization for X-ray tube and the detector. 10–80° (2θ) were scanned with a 5°/min scanning rate. The generator voltage was 40 kV and the current was 40 mA. QEMSCAN analysis was conducted by a Quanta 650-F (Thermo Science) SEM platform mounted with two DualXFlash 5030 (Bruker) energy dispersive detectors. Furthermore, the system mounts a 4 quadrant BSE detector for acquiring images. The measurements were performed using an acceleration voltage of 25 KeV and a fixed sample current of 10 nA.

## Supplementary information

Supplementary Information

## Data Availability

The datasets generated during and/or analysed during the current study are available from the corresponding author on reasonable request.
