# Supplementary material for: Synthesis of Scandium Phosphate after Peroxide Assisted Leaching of Iron Depleted Bauxite Residue (Red Mud) Slags
Source: Sci Rep. 2019 Aug 14;9:11803. doi: 10.1038/s41598-019-48390-z (PMC6694154; doi:10.1038/s41598-019-48390-z)
Supplement: Supplementary file 1 — Supplementary Information [file 41598_2019_48390_MOESM1_ESM.docx]

**Supplementary Information**

**Synthesis of Scandium Phosphate after Peroxide Assisted Leaching of Fe-Depleted Bauxite Residue (Red Mud) Slags**

Bengi Yagmurlu^1,2,*,+^, Gözde Alkan^1,+^, Buhle Xakalashe^1,+^, Claudia Schier^1^, Lars Gronen^3^, Ichiro Koiwa^4^, Carsten Dittrich^2^, Bernd Friedrich^1^

**^1^** IME- Process Metallurgy and Metal Recycling, RWTH Aachen University, Germany

^2^ MEAB Chemie Technik GmbH, Germany

^3^IML- Chair of Applied Mineralogy and Economic Geology, RWTH Aachen University, Germany

^4^College of Science and Engineering, Kanto Gakuin University, Japan

* Corresponding author

+ These authors contributed equally to this work.

Author to whom correspondence should be addressed:

e-mail: bengi@meab-mx.com

bengiyagmurlu@gmail.com

Address: Dennewartstr. 25, 52068 Aachen (Germany)


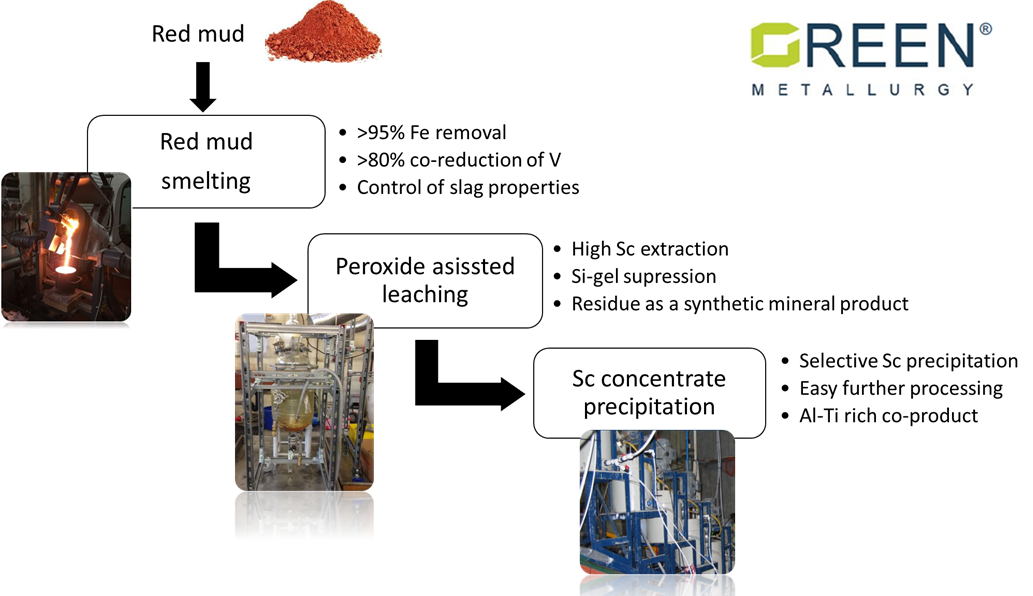


**Figure S1.** A complete utilization process designed by IME-RWTH Aachen & MEAB





**Figure S2.** Quantitative mineralogical distribution of the synthesized slags


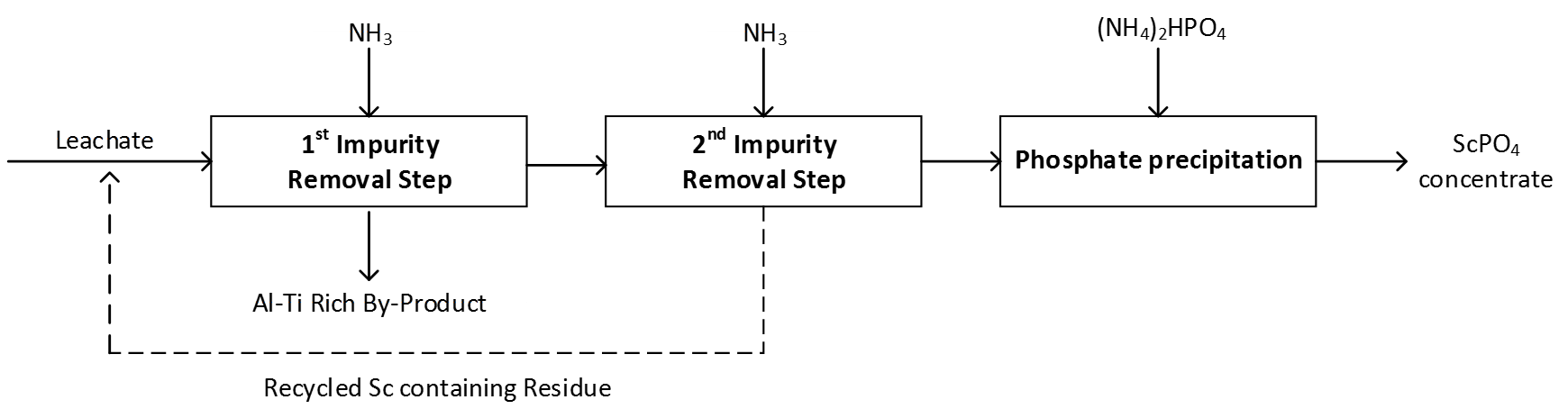


**Figure S3.** Triple-staged successive precipitation process flow diagram to synthesize Sc phosphate

**Table S1.** Composition of the PLS resulting from leaching of fast cooled basic slag with 2.5 M H_2_SO_4_-2.5 M H_2_O_2_

| **Major Elements** | **g/L** | **Minor Elements** | **mg/L** |
| --- | --- | --- | --- |
| Al | 18.6 | Sc | 16 |
| Fe | 1.7 | Ce | 16 |
| Ti | 3.1 | Y | 9 |
| Ca | 0.6 | La | 4 |
| Si | 0.1 | Nd | 4 |
|  |  | U | 2 |
|  |  | Th | 11 |
